# Preventive Effects of Probiotic Formula on Metabolic Stress Associated Physical Fatigue in Forced Swimming and LPS-Induced Mouse Models

**DOI:** 10.4014/jmb.2603.03034

**Published:** 2026-04-10

**Authors:** Jae Gwang Song, Hyun Jin Bae, Dong Hwan Lee, Joeun Seo, Bomi Lee, Kum-Joo Shin, Eui-Chun Chung, Jeongwook Lee, Hyung Wook Kim, Nam Su Oh

**Affiliations:** 1 Department of Integrative Bioscience and Biotechnology, Sejong University, Seoul 05006, Republic of Korea; 2 Department of Food and Biotechnology, Korea University, Sejong 30019, Republic of Korea; 3 R&D Center, Hecto Healthcare Co. Ltd., Seoul 06142, Republic of Korea

**Keywords:** Myalgic encephalomyelitis/chronic fatigue syndrome, Lactate metabolism, Inflammation, Metabolic stress, Chronic fatigue, Probiotics

## Abstract

Myalgic encephalomyelitis/chronic fatigue syndrome (ME/CFS) is a complex disorder characterized by persistent fatigue and post-exertional symptom exacerbation, frequently associated with immune and metabolic disturbances. To evaluate the therapeutic potential of a probiotic formula, HH-205M, we employed a composite mouse model combining forced swimming stress (FSS) and repeated lipopolysaccharide (LPS) administration. FSS–LPS exposure induced pronounced fatigue-like phenotypes, including reduced physical endurance capacity in treadmill and weight-loaded swimming tests, delayed recovery in post-swim grooming behavior, and increased thermal pain sensitivity. These behavioral impairments were accompanied by elevated serum creatine kinase (CK), lactate dehydrogenase (LDH), and lactate levels, indicating systemic metabolic stress. At the tissue level, FSS–LPS increased lipid peroxidation and upregulated pro-inflammatory cytokine expression while suppressing antioxidant gene expression in the gastrocnemius muscle. Furthermore, expression of lactate-related genes, *Hcar1* (GPR81) and *Slc16a1* (MCT1), was reduced, suggesting disruption of lactate transport and sensing pathways under chronic stress and inflammatory conditions. HH-205M supplementation attenuated the elevations in circulating fatigue-related biomarkers, moderated oxidative and inflammatory responses, and restored *Hcar1* and *Slc16a1* expression. These molecular changes were paralleled by improvements in endurance performance and nociceptive sensitivity. HH-205M administration was also associated with distinct shifts in gut microbial composition, including enrichment of *Akkermansia* and *Bacteroides* and reduced relative abundance of *Alistipes*. Collectively, these findings indicate that the FSS–LPS composite model recapitulates inflammation-associated metabolic disturbances relevant to fatigue-like conditions and that HH-205M administration is associated with concurrent improvements in behavioral and molecular parameters in this model.

## Introduction

Myalgic encephalomyelitis/chronic fatigue syndrome (ME/CFS) is a debilitating multisystem disorder characterized by persistent and profound fatigue that is not alleviated by rest [1, 2]. This condition is accompanied by a range of incapacitating symptoms, including post-exertional malaise, unrefreshing sleep, cognitive impairment, and widespread musculoskeletal pain [3, 4]. The complex symptomatology and lack of definitive biomarkers make the diagnosis and treatment of ME/CFS challenging, imposing a substantial burden on patients and society [5, 6].

Although the precise etiology of ME/CFS remains unclear, accumulating evidence suggests that chronic fatigue is associated with immune dysregulation and metabolic disturbances, with systemic inflammation as a prominent feature [6, 7]. Elevated circulating pro-inflammatory cytokines, including interleukin-1β (IL-1β), interleukin-6 (IL-6), and tumor necrosis factor-α (TNF-α), have been reported in patients with ME/CFS [8-10]. In parallel, alterations in skeletal muscle energy metabolism have been observed in ME/CFS [7]. Patients exhibit reduced oxidative phosphorylation capacity and a greater reliance on glycolytic metabolism [11, 12], accompanied by abnormal lactate accumulation in blood or cerebrospinal fluid even at rest or under low exertion [13, 14]. Inflammatory activation within skeletal muscle is thought to contribute to this metabolic shift by increasing oxidative stress and disrupting mitochondrial oxidative pathways, including pyruvate utilization and oxidative phosphorylation [15]. These findings collectively suggest a disturbance in metabolic flexibility, which may contribute to early fatigue onset and reduced endurance capacity.

A key limitation of ME/CFS research is that animal models often focus on a single stressor, such as forced swimming stress (FSS) for physical fatigue or lipopolysaccharide (LPS) injection for immune challenges [16, 17]. However, this single-stressor approach fails to fully recapitulate the multifaceted pathology of ME/CFS, which involves physical exhaustion and chronic immune activation [18, 19]. Therefore, composite models have been proposed to more closely reflect the multifactorial nature of ME/CFS. The FSS + LPS model, which combines chronic physical exertion with a systemic inflammatory challenge, has been proposed as a more robust platform for studying the complex interplay of fatigue, pain, and inflammation [18].

Recently, probiotics have emerged as potential modulators of immune dysregulation, oxidative stress, and metabolic disturbances, with accumulating evidence demonstrating their capacity to regulate inflammatory responses and improve host metabolic homeostasis [21, 22]. The probiotic formula HH-205M (previously known as SLAB51), (previously known as SLAB51) has been reported to exert anti-inflammatory and antioxidant effects and to influence metabolic and oxygen-related parameters in various experimental models and clinical studies [23, 24]

In this study, we employed an FSS–LPS composite mouse model to investigate fatigue-like phenotypes and to evaluate the effects of the HH-205M on behavioral performance, inflammatory responses, oxidative stress, and lactate-related metabolic parameters.

## Material and Methods

### Muscle Atrophy and Inflammatory Response in C2C12 Myotubes

The probiotic formulation, HH-205M, was provided by Hecto Healthcare (Republic of Korea) and stored at 4°C. HH-205M is a probiotic mixture consisting of *S. thermophilus* (CNCM I-5570), *Bifidobacterium animalis* subsp. *lactis* (CNCM I-5571), *Bifidobacterium animalis* subsp. *lactis* (CNCM I-5572), *L. acidophilus* (CNCM I-5567), *L. helveticus* (CNCM I-5573), *L. paracasei* (CNCM I-5568), *L. plantarum* (CNCM I-5569), and *L. brevis* (CNCM I-5566), previously described as SLAB51. HH-205M was inoculated into Dulbecco's Modified Eagle's Medium high glucose (DMEM, high glucose; Biowest, France) at 1 × 10^9^ CFU/ml and cultured for 24 h at 37°C in a humidified atmosphere containing 5% CO_2_. The supernatant was then collected and neutralized with 0.1 N NaOH, followed by filtration (0.22 μm pore size) to produce conditioned media (CM).

Differentiated C2C12 myotubes (American Type Culture Collection, USA) were used to establish an oxidative injury model. To induce oxidative stress, cells were treated with 2 mM hydrogen peroxide (H_2_O_2_) and simultaneously treated with HH-205M conditioned media (CM; 1%, 5%, or 10%, v/v) for 24 h. After treatment, cell viability was assessed using the CellTiter 96^®^ Non-Radioactive Cell Proliferation Assay (Promega, USA) according to the manufacturer’s instructions.

To induce myotube atrophy, differentiated C2C12 myotubes were treated with dexamethasone (DEX; 100 μM) for 24 h. Morphological changes were evaluated by measuring myotube thickness/diameter. For diameter measurements, representative bright-field images were acquired under identical exposure and magnification settings. Myotube diameter was quantified using ImageJ software (National Institutes of Health, USA) by measuring the width at multiple points along each myotube, and the results were summarized per condition (*e.g.*, median value per well/condition). To minimize sampling bias, at least ~30 myotubes per condition were analyzed, and the mean/median diameter was used for statistical comparisons.

To establish an inflammatory response model, differentiated C2C12 myotubes were stimulated with H_2_O_2_ (2 mM) in the presence or absence of CM (1%, 5%, or 10%, v/v) for 24 h. After stimulation, culture supernatants were collected for cytokine analysis. The concentrations of TNF-α and IL-6 in the culture supernatants were determined using commercial ELISA kits (Cusabio, USA) according to the manufacturer’s instructions.

### Animals and Tissue Collection

Male C57BL/6 mice (4 weeks old) were purchased from Daehan Biolink (Republic of Korea). Mice were maintained in a controlled environment (temperature: 22 ± 2°C, humidity: 50 ± 10%) under a 12 h light/dark cycle with ad libitum access to standard laboratory chow and water. After all behavioral tests were completed, the mice were anesthetized using 5% isoflurane (Hana Pharm Co. Ltd., Republic of Korea). To investigate the underlying mechanisms of the behavioral changes, we collected blood samples, skeletal muscle, and colon. Additionally, fecal samples were collected for microbiome analysis. Collected samples were stored at -80°C until analysis. All animal experiments were performed in accordance with the guidelines of the Institutional Animal Care and Use Committee of Sejong University (approval no. SJ-20230110-01).

### Experimental Design

After acclimation, the mice were randomly assigned to one of the three experimental groups (n = 15 per group): CTL-Saline: control mice treated with saline; CFS-Saline: CFS model mice treated with saline; or CFS-HH-205M: CFS model mice treated with HH-205M. For administration, HH-205M was freshly suspended in sterile saline prior to use. The HH-205M suspension was administered daily via oral gavage at a dose of 2 × 10^9^ CFU/day. The vehicle control groups (CTL-Saline and CFS-Saline) received equivalent volumes of sterile saline. HH-205M administration began 2 weeks before the onset of the CFS induction protocol (Week 0–2, pretreatment period) and continued daily throughout the 7-week CFS induction and behavioral testing period (Week 2–9), resulting in a total administration period of 9 weeks.

### CFS Model

To establish a CFS-like model, the mice were subjected to a combined stress protocol consisting of FSS and LPS administration.

### FSS

To induce physical fatigue, mice were subjected to FSS for 20 min/day for 7 weeks (Week 2–9), as previously described, with slight modifications [25]. FSS was conducted in transparent acrylic cylinders (250 mm height, 120 mm length, 120 mm width) filled with water (20 cm depth) maintained at 20 ± 0.5°C, where mice were individually placed in each cylinder. Daily FSS exposure was continued during the behavioral testing period (Week 6–9) to maintain CFS.

### LPS administration

To induce systemic immune disturbances and inflammation, mice received intraperitoneal injections of LPS (*Escherichia coli* O111:B4, Sigma-Aldrich, USA, Cat. No. L2630) for 4 weeks (Week 2–6), as previously described with slight modifications [26]. LPS was administered according to a dose-escalation schedule: 0.208, 0.415, and 0.83 mg/kg (3 days/week) during Weeks 2–4, and 0.208, 0.415, 0.83, and 0.83 mg/kg (4 days/week) during Weeks 4–6. The control group (CTL-Saline) received intraperitoneal injections of sterile saline following the same schedule.

### Behavioral Analysis

**Treadmill Test (TMT).** The TMT was performed to evaluate exercise endurance as previously described with slight modifications [27, 28]. Mice were tested on a motorized treadmill with a 5° incline, and a mild electric shock (0.2 mA) was delivered through the rear grid throughout the 5-day protocol. The procedure comprised habituation and progressive training, followed by a final test (Day 5). The training intensity was incrementally increased as follows: Day 1 (0 m/min for 3 min, then 6 m/min for 5 min), Day 2 (6–10 m/min for 10 min), Day 3 (6–15 m/min for 20 min), and Day 4 (6–20 m/min for 30 min). The maximum speed reached on Day 4 was defined as the early exhaustion threshold, and mice failing to reach this target speed during the final test (Day 5) were classified as exhibiting early exhaustion. On the test day (Day 5), the treadmill speed was progressively increased from 6 to 25 m/min over a 30-min period. The test was terminated when the mouse remained on the shock grid for five consecutive seconds (defined as exhaustion) or reached a maximum duration of 30 min. The total distance travelled, time to exhaustion, and the rate of early exhaustion were recorded and analyzed using ANY-maze 7.51 software.

**Weight-loaded Forced Swimming Test (w.l.FST).** Physical endurance was assessed using the w.l.FST, as previously described with slight modifications [29]. A weight corresponding to 10% of the mouse body weight was attached to the tail. Mice were placed in a water-filled glass cylinder (26 cm height, 18 cm diameter, and water depth of 20 cm at 25 ± 1°C). The latency to exhaustion, defined as the time until the mouse failed to rise to the surface for seven consecutive seconds, was measured. To assess the potential confounding effect of body weight on the latency to exhaustion, simple linear regression analysis was performed.

**Post-swim Grooming Test (PSGT).** The PSGT was performed to assess fatigue-induced changes in self-care behavior, specifically targeting post-exertional malaise-like symptoms characterized by delayed recovery following physical stress [17]. Immediately after the FSS, the mice were gently individually placed in a transparent polypropylene cylinder (80 mm height, 120 mm diameter). The self-grooming activity was recorded using a video camera (C920; Logitech, Switzerland) for subsequent analyses. Grooming behavior was defined as rubbing the face with the forepaws, licking the paws, and scratching or licking the body coat. The time spent grooming was measured over a period of 20 min immediately following the swim. Comparisons between the two CFS groups were performed using an unpaired Student's t-test.

**Tail immersion test.** Thermal pain sensitivity (nociception) was measured by immersing the distal 3–4 cm of the tail in a thermostatically controlled water bath maintained at 49 ± 0.5°C , as previously described with slight modifications [30]. The latency to the first rapid tail flick was recorded using a video camera (C920; Logitech, Switzerland). A cutoff time of 10 s was used to prevent tissue damage.

### Biochemical and Molecular Analysis

**Serum analysis.** Blood samples were collected from anesthetized mice via cardiac puncture, after which the animals were sacrificed. The blood in serum separation tubes was centrifuged at 2,500 × g for 20 min at 4°C. Serum was collected and used for biochemical analysis. CK and LDH concentrations were measured according to the International Federation of Clinical Chemistry and Laboratory Medicine procedures [31, 32], and blood lactate concentrations were determined using an enzymatic method [33] with an automatic biochemical analyzer (Hitachi-7180, Japan).

**Measurement of lipid peroxidation.** Malondialdehyde (MDA) levels were quantified using a slightly modified version of the method described by Reilly and Aust [34]. Gastrocnemius muscle tissues were homogenized in ice-cold 1× phosphate-buffered saline (pH 7.4) to obtain 10% (w/v) homogenates. The homogenates were mixed with 1× thiobarbituric acid/trichloroacetic acid/HCl reagent, prepared with a four-fold dilution of a 4× stock solution (0.37% thiobarbituric acid, 15% trichloroacetic acid, and 0.25 N HCl), and incubated in a boiling water bath for 15 min. Then, the samples were cooled at 4°C for 5 min and centrifuged at 10,000 × g for 10 min at 4°C. The amount of MDA-TBA adduct was measured at 535 nm using a microplate spectrophotometer (Epoch 2; BioTek, USA). A standard curve was constructed using 1,1,3,3-tetramethoxypropane (TMP) solutions.

**Quantitative Real-time reverse transcription PCR (qRT-PCR) analysis.** To examine the effects of HH-205M administration on the mRNA expression of genes related to lactate metabolism (hydroxycarboxylic acid receptor 1; *Hcar1* and the solute carrier family 16 (monocarboxylic acid transporters), member 1; *Slc16a1*), antioxidant defense (superoxide dismutase 1; *Sod1* and glutathione peroxidase-1; *Gpx1*), and inflammation (tumor necrosis factor-α; *Tnf*, interleukin-1β; *Il1b*, and interleukin-6; *Il6*, and the pro-inflammatory enzymes, inducible nitric oxide synthase; *Nos2* and cyclooxygenase-2; *Ptgs2*), qRT-PCR was performed. Total RNA was isolated from the gastrocnemius muscle, and colon using the TRIzol reagent (Invitrogen, USA) according to the manufacturer’s instructions. RNA concentration and purity were assessed using a microplate spectrophotometer (Epoch 2; BioTek). Complementary DNA was synthesized from RNA using ReverTra Ace qPCR RT Master Mix (TOYOBO, Japan). qRT-PCR was performed on an Applied Biosystems QuantStudio^TM^ 3 Real-Time PCR System (Thermo Fisher Scientific) using THUNDERBIRD^®^ Next SYBR^TM^ qPCR Mix (TOYOBO). The amplification protocol consisted of an initial denaturation at 95°C for 30 s, followed by 40 cycles of denaturation at 95°C for 5 s and annealing/extension at 60°C for 30 s. Gene expression levels were normalized to *Gapdh* and calculated using the 2^-ΔΔCt^ method. Supplementary Table S1 lists the primer sequences used for amplification.

### Microbiome Analysis

**DNA extraction.** Mouse fecal samples were stored at -80°C until DNA extraction. Following the manufacturer's recommendations, total bacterial gDNA was extracted using the Maxwell^®^ RSC PureFood GMO and Authentication Kit (Promega, USA). The concentration of bacterial DNA was measured using the QuantiFluor^®^ ONE dsDNA System (Promega). The DNA samples were stored at -20°C until further analysis.

**16S rRNA amplification and MiSeq sequencing.** The V3-V4 variable region of the *16S* rRNA gene was amplified from DNA extracts using a *16S* metagenomic sequencing library (Illumina, USA). Two PCRs were performed on template DNA. Initially the DNA was amplified with primers specific to the V3-V4 region of the 16S rRNA gene, which also incorporates the Illumina overhang adaptor (Forward primer 5'-TCGTCGGCAGCGTAGATGTGTAAGAGACGCCTCGGGNGCWGCAG-3'; reverse primer 5'-GTCTCGTGGGCTCGGAGATGTGTATAA GAGACAGGCTACHVGGT-TCAATCC-3'. Each PCR reaction contained DNA template (~10–12 ng), 5 μl of forward primer (1 μM), 5 μl of reverse primer (1 μM), 12.5 μl of 2 × Kapa HiFi Hotstart ready mix (Kapa Biosystems, USA), and PCR grade water to a final volume of 25 μl. PCR amplification was carried out as follows: heated lid 110°C, 95°C for 3 mins, 25 cycles of 95°C for 30s, 55°C for 30s, 72°C for 30s, 72°C for 5 mins, and held at 4°C. PCR products were visualized using gel electrophoresis (1 × TAE buffer, 2% agarose, 100 V). Successful PCR products were purified using an AMPure XP magnetic bead-based purification kit (Beckman Coulter, UK) and run on an Agilent Bioanalyzer for quality analysis.

The second PCR reaction was completed on the purified DNA (5 μl) to index each of the samples. Two indexing primers (Nextera XT indexing primers, Illumina) were used per sample. Each PCR reaction contained 5 μl of index 1 primer (N7xx), 5 μl of index 2 primer (S5xx), 25 μl of 2× Kapa HiFi Hot Start Ready mix, and 10 μl of PCR grade water. PCR was performed as described above, but only eight amplification cycles were completed instead of 25. Successful PCR products were purified using the AMPure XP magnetic bead-based purification kit (Beckman Coulter). The purified products were quantified using a QuantiFluor^®^ ONE dsDNA System (Promega) and run on the Agilent Bioanalyzer. The sample pool (4 nM) was denatured with 0.2 N NaOH, then diluted to 20 pM, and combined with 10% (v/v) denatured 8 pM PhiX, prepared following Illumina guidelines. Samples were sequenced on a MiSeq sequencing platform using a 2 × 300 cycle V3 kit, following standard Illumina sequencing protocols.

**Data processing for analysis of 16S rRNA sequencing data.** The sequenced dataset was processed using QIIME2 package (version 2023.05). All data handling was performed using QIIME2 plugins. After creating the data artifact in QIIME2, forward and reverse adapters were detected and removed from demultiplexed samples via “cutadapt trim-paired.” Paired-end reads were then combined using “vsearch merge-pairs,” and low-quality bases were filtered out with “quality-filter q-score.” Denoising was achieved using a Deblur algorithm by applying a trim length of 251. For taxonomic classification, sequences were aligned with the SILVA database (“silva-138.1-99-nb-classifier.qza”, https://www.arb-silva.de/archive/release_138.1/Exports) to assign taxonomies at the phylum, class, order, family, genus, and species levels. To isolate the bacterial sequences, the taxa plugin was used to filter out non-bacterial sequences (archaea, eukaryotes, etc.). The samples with a total frequency of zero were excluded. For normalization of sampling depth, the “feature-table rarefy” function was applied, using a minimum read count. Taxon abundance per level was obtained by collapsing taxa with the “taxa collapse” function, set to a 97% sequence identity threshold for genus-level classification. A relative abundance table was generated by calculating the frequency of each taxon in relation to the total frequency in each sample through “feature-table relative-frequency.”

The diversity plugin in QIIME2 was used to assess alpha diversity, which represents intra-community diversity, and beta diversity, which represents inter-community diversity. Alpha diversity was estimated using multiple metrics, including the Observed ASVs, Shannon index, Simpson index, and Faith’s Phylogenetic Diversity indices. Beta diversity was analyzed using the Bray–Curtis metric to generate a distance matrix, which was visualized using principal coordinate analysis. To evaluate whether the microbial community composition differed significantly between groups, permutational multivariate analysis of variance was performed on the Bray–Curtis distance matrix using the adonis2 function in the vegan R package with 10,000 permutations. To assess the assumption of homogeneity of multivariate dispersions, a permutational analysis of multivariate dispersions was conducted using the betadisper function, followed by a permutest in vegan, with 10,000 permutations. Additionally, analysis of the composition of microbiome-bias correction was conducted in QIIME2 to identify significant differences in the bacterial taxa.

### Statistical Analysis

All statistical analyses were performed using GraphPad Prism 10.5 (GraphPad Software, USA) and all parametric data are presented as the mean ± standard error of the mean (SEM), while non-parametric data are presented as the median with interquartile range (IQR). Normality of data distribution was assessed using the Shapiro-Wilk test. Homogeneity of variances was evaluated using the Brown–Forsythe test. For multi-group comparisons, data satisfying normality and variance-homogeneity assumptions were analyzed by one-way analysis of variance (ANOVA) followed by Dunnett’s post-hoc test. For datasets showing heterogeneity of variance, group differences were evaluated using Welch’s one-way ANOVA, with Dunnett’s T3 procedure used. Non-normally distributed data were analyzed using the non-parametric Kruskal–Wallis test followed by Dunn’s post-hoc test with Benjamini-Hochberg correction to control the false discovery rate (FDR). Statistical significance was defined as *p* < 0.05 (or *q* < 0.05 for FDR-corrected data).

## Results

### HH-205M Treatment Protects C2C12 Myotubes from Oxidative Stress, Muscle Atrophy, and Inflammation

**Fig. 1:**
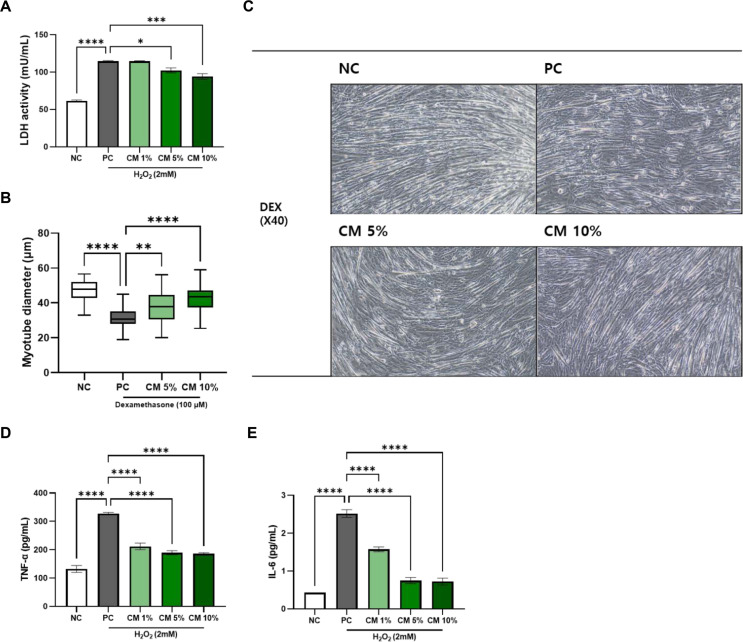
Protective effects on oxidative stress, muscle atrophy, and inflammatory response of HH-205M treatment in C2C12 myotubes. (**A**) LDH levels in the culture supernatant. (**B**) Quantification of myotube diameter and (**C**) representative morphological images (magnification, ×40) following DEXinduced atrophy. (**D, E**) Secretion levels of pro-inflammatory cytokines, TNF-α and IL-6, determined by ELISA. For A, D and E, data are presented as mean ± SEM. For B, data presented as median with IQR and minimum-maximum values. Significance is denoted as *p < 0.05, **p< 0.01, ***p < 0.001 and ****p < 0.0001 compared to the PC group. LDH, lactate dehydrogenase, DEX, dexamethasone; IQR, interquartile range.

To evaluate membrane damage, LDH release into the culture supernatants was quantified. Prior to the main challenge, treatment with CM alone (1%, 5%, and 10%, v/v) in the absence of H_2_O_2_ induced no detectable cytotoxicity. H_2_O_2_ exposure induced a robust increase in LDH release, indicating muscle cell membrane injury. However, CM attenuated this release in a concentration-dependent manner: LDH release showed a significant decrease at 5% (*p* < 0.05) and 10% CM (*p* < 0.0001) compared to the H_2_O_2_ -only treated group (Fig. 1A).

To model muscle atrophy, differentiated C2C12 myotubes were treated with 100 μM DEX for 24 h, which resulted in a clear reduction in myotube diameter. Co-treatment with CM (5% and 10%) attenuated the DEX-induced decrease in myotube thickness in a dose-dependent manner, with 10% CM showing a more pronounced protective effect (Fig. 1 B and 1C).

To evaluate the anti-inflammatory potential of CM (1%, 5%, and 10%, v/v), differentiated C2C12 myotubes were treated with H_2_O_2_ (2 mM). CM significantly reduced pro-inflammatory cytokine production in the culture supernatant. Both TNF-α and IL-6 levels were significantly decreased starting at 1% CM, with further reductions observed at higher concentrations (Fig. 1D and 1E). These results suggest that CM possesses potent anti-inflammatory properties that may contribute to its muscle-protective effects.

### HH-205M Treatment Attenuated FSS-LPS Induced Increases in Serum Fatigue-Related Biomarkers

**Fig. 2:**
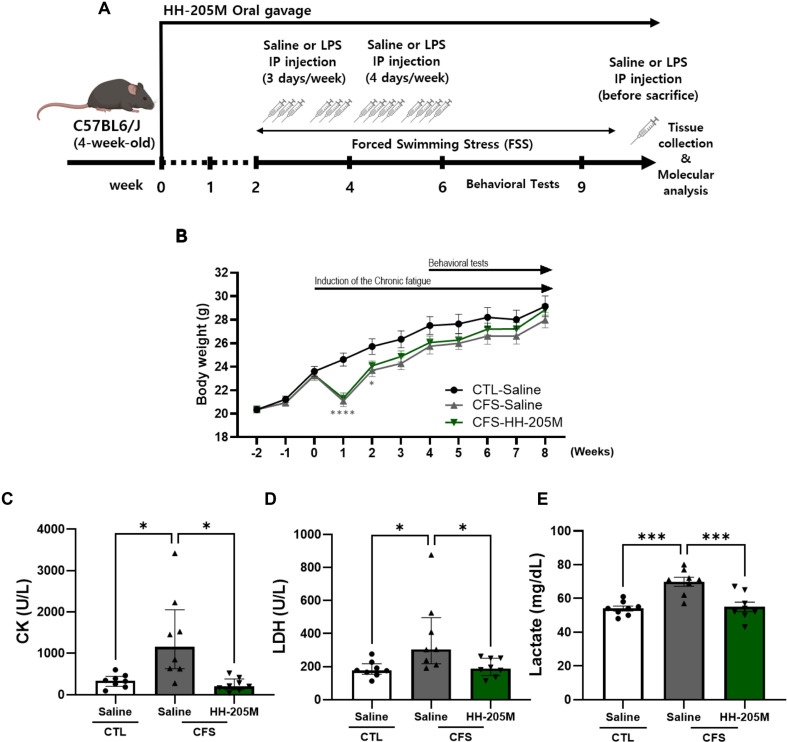
Experimental design of mouse model and change of biochemical markers in serum. (**A**) Schematic representation of the study protocol describing the 9-week experimental schedule. The study consisted of a 2-week pretreatment period followed by a 7-week induction period involving LPS injection and forced swimming stress (FSS). HH-205M or saline was administered daily via oral gavage. Behavioral tests were conducted during weeks 6–9. (**B**) Body weight changes monitored weekly throughout the experiment (n = 12–15 per group). (C–E) Serum levels of creatine kinase (CK), lactate dehydrogenase (LDH), and lactate were measured. Parametric data are presented as the mean ± SEM and non-parametric data are presented as the median with IQR (n = 8 per group). For **C** and **D**, data were analyzed using the Kruskal–Wallis test followed by Dunn’s post-hoc test with Benjamini-Hochberg correction, and asterisks indicate discoveries (*q < 0.05). For E, data were analyzed using the one-way ANOVA, and significance is denoted as ***p < 0.001. LPS, lipopolysaccharide

The experimental protocol was conducted over 9 weeks (Fig. 2A). The study comprised a 2-week pretreatment period, followed by a 7-week CFS induction period, during which FSS and LPS were administered. Behavioral tests were conducted during Week 6-9.

We validated the systemic effects of the CFS model. Body weights were monitored throughout the 9-week study period (Fig. 2B). Body weight changes over time were analyzed using two-way ANOVA followed by Dunnett’s post-hoc test. During the initial induction phase, the CFS groups exhibited a transient but significant decrease in body weight compared with that in the CTL-Saline group. However, the body weight in the CFS groups recovered to levels comparable to those in the CTL-Saline group, with no significant differences observed among the groups at the end of the behavioral tests.

To evaluate alterations of systemic biomarkers, we quantified serum CK, LDH, and lactate levels. The CFS-Saline group exhibited significant increases in CK (*q* < 0.05), LDH (*q* < 0.05), and lactate (*p* < 0.001) levels compared with those of the CTL-Saline group, indicating pronounced systemic metabolic stress under combined FSS-LPS exposure (Fig. 2C–2E). In contrast, HH-205M supplementation markedly attenuated these elevations, indicated by a reduction in all three markers to control levels (CK, LDH: *q* < 0.05; lactate: *p* < 0.001 vs. CFS-Saline). These findings suggest that HH-205M was associated with reduced circulating metabolic stress markers that are elevated under conditions of chronic stress and inflammation.

### HH-205M Treatment Ameliorates Deficits in Loaded Physical Endurance, Fatigue Recovery and Pain Hypersensitivity

**Fig. 3:**
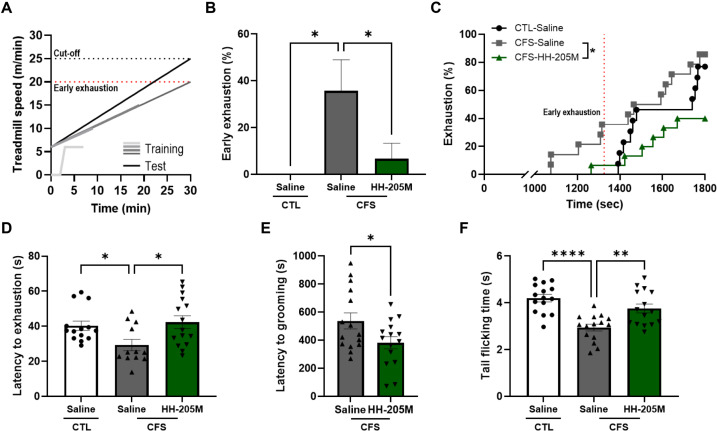
Effect of HH-205M treatment on physical endurance, fatigue-related behavioral deficits, and pain hypersensitivity. (**A**) Protocol for the TMT with progressive intensity. (**B**) Rate of early exhaustion during the TMT. (**C**) Exhaustion survival plot from the TMT. (**D**) Latency to exhaustion in the w.l.FST. (**E**) Latency to initiate grooming behavior in the PSGT. (**F**) Latency to tail flick in the TIT. Parametric data are presented as the mean ± SEM and non-parametric data are presented as the median with IQR (n = 12–15 per group). For B and D, data were analyzed using the Kruskal–Wallis test followed by Dunn's post hoc test with Benjamini–Hochberg correction, and asterisks indicate discoveries (*q < 0.05). For C, survival rates were analyzed using the Kaplan-Meier method, and group differences were determined by the Log-rank (Mantel-Cox) test. For E, data were analyzed using unpaired Student's t-test for comparisons between the two CFS groups, while F was analyzed using one-way ANOVA with Dunnett's post hoc test. Data are presented as the mean ± SEM (n = 12–15 per group). For parametric analyses, significance is denoted as *p < 0.05, **p < 0.01, and ****p < 0.0001. TMT, treadmill test; w.l.FST, weight-loaded forced swimming test; PSGT, post-swim grooming test; TIT, tail immersion test

We evaluated the effect of HH-205M on loaded physical endurance using the TMT and w.l.FST. The TMT protocol involved a progressive increase in speed (Fig. 3A). During the 4 days of training, the running speed and duration progressively increased, and maximum speed on Day 4 was defined as the early exhaustion point. The CFS-Saline group exhibited an early exhaustion rate of 35.7%, which was significantly higher than that of the CTL-Saline group (Fig. 3B, *q* < 0.05). The CFS-HH-205M group showed a significant reduction in the early exhaustion rate to 6.7% compared with that of the CFS-Saline group (*q* < 0.05). This improvement was also observed in the exhaustion survival plot (Fig. 3C, *p* < 0.05). Furthermore, the final exhaustion rate of the HH-205M-treated group was lower than that of the Saline-treated group and remained below 50%.

This improvement in endurance was confirmed using the w.l.FST. As body weight can influence swimming endurance [35, 36], we examined the correlation between body weight and raw exhaustion time. However, the regression model was not statistically significant (F_(1, 39)_ = 2.277, *p* = 0.1393). Consequently, the raw latency to exhaustion was analyzed without covariate adjustment (Fig. 3D). The CFS-Saline group showed a shorter latency to exhaustion than that of the CTL-Saline group (*q* < 0.05), whereas the CFS-HH-205M group exhibited a significantly longer latency to exhaustion than that of the CFS-Saline group (*q* < 0.05).

Next, we assessed other key symptoms of CFS. To evaluate fatigue-induced changes in self-care behavior, we used the PSGT (Fig. 3E). The PSGT, which was performed immediately after an FSS session, measures the latency for a mouse to initiate self-grooming, with a longer latency indicating greater fatigue [37]. The CFS-HH-205M group exhibited a significantly shorter latency to initiate grooming behavior compared with that of the CFS-Saline group (*p* < 0.05), suggesting an improvement in recovery from fatigue.

Finally, we assessed pain sensitivity as a key comorbid symptom of chronic fatigue using the tail immersion test (Fig. 3F) [18]. The CFS-Saline group exhibited a significantly shorter tail-flicking time compared with that of the CTL-Saline group (*p* < 0.0001), confirming the robust induction of thermal hyperalgesia. The CFS-HH-205M group showed a significantly longer tail-flicking latency than that of the CFS-Saline group (*p* < 0.01), suggesting that HH-205M supplement effectively ameliorated fatigue-associated pain hypersensitivity.

### HH-205M Treatment Modulates Lactate Transport, Oxidative Stress, and Inflammatory Markers in the Gastrocnemius Muscle

**Fig. 4:**
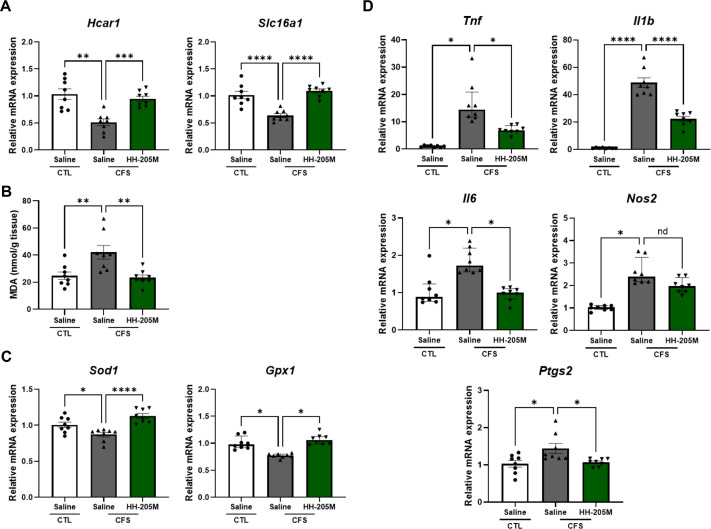
Effects of HH-205M treatment on oxidative stress, inflammatory gene and *Hcar1*/*Slc16a1* expression in gastrocnemius muscle. (**A**) mRNA expression of *Hcar1* and *Slc16a1* was determined. (**B**) MDA content was quantified. (**C**) mRNA expression of *Sod1* and *Gpx1* was determined. (**D**) Relative mRNA expression of the pro-inflammatory cytokines, *Tnf*, *Il1b*, and *Il6*, and the pro-inflammatory enzymes, *Nos2* and *Ptgs2*, was determined. Parametric data are presented as the mean ± SEM and non-parametric data are presented as the median with IQR (n = 8 per group). For A (*Hcar1*) and D (*Il1b*), data were analyzed using Welch’s one-way ANOVA followed by Dunnett's multiple comparisons test. For A (*Slc16a1*), B and D (*Ptgs2*), data were analyzed using the one-way ANOVA with Dunnett's post hoc test. For B and D (*Tnf*, *Il6*, *Nos2*), data were analyzed using the Kruskal–Wallis test followed by Dunn’s post-hoc test with Benjamini–Hochberg correction, and asterisks indicate discoveries (*q < 0.05). For parametric analyses, significance is denoted as *p < 0.05, **p < 0.01, ***p < 0.001 and ****p < 0.0001.

We assessed the gene expression of hydroxycarboxylic acid receptor 1 (*Hcar1*; GPR81) and the solute carrier family 16 (monocarboxylic acid transporters), member 1 (*Slc16a1*; MCT1) in the gastrocnemius muscle (Fig. 4A). The CFS-Saline group showed a significant reduction in *Hcar1* (*p* < 0.01) and *Slc16a1* (*p* < 0.0001) expression relative to those in the CTL-Saline group, reflecting a marked disruption of the lactate transport-sensing system. However, HH-205M supplementation significantly restored the expression of *Hcar1* (*p* < 0.001 vs. CFS-Saline) and *Slc16a1* (*p* < 0.0001 vs. CFS-Saline).

To assess oxidative stress under CFS-like conditions, MDA levels and antioxidant enzyme expression were quantified in the gastrocnemius muscle (Fig. 4B and 4C). The CFS-Saline group showed a significant increase in MDA (*p* < 0.01 vs. CTL-Saline), indicating enhanced lipid peroxidation. Gene expression levels of *Sod1* and *Gpx1* were significantly reduced in the CFS-Saline group compared with those in the CTL-Saline group (*Sod1*: *p* < 0.05; *Gpx1*: *q* < 0.05), suggesting impaired removal of superoxide radicals and hydrogen peroxide. However, HH-205M supplementation modulated these oxidative changes. MDA accumulation was significantly reduced (*p* < 0.01 vs. CFS-Saline) in the CFS-HH-205M group. Consistently, *Sod1* and *Gpx1* expression levels were significantly increased in the CFS-HH-205M group (*Sod1*: *p* < 0.0001; *Gpx1*: *q* < 0.05). These findings indicate that HH-205M modulates lipid peroxidation and supports enzymatic antioxidant defenses in skeletal muscle under CFS-like conditions.

The FSS-LPS exposure induced inflammation in the muscle. As shown in Fig. 4D, the CFS-Saline group exhibited a significant increase in the mRNA expression of pro-inflammatory cytokines (*Tnf*, *Il1b*, and *Il6*) and inflammatory enzymes (*Nos2* and *Ptgs2*) in the gastrocnemius muscle (*Tnf*, *Il6*, *Nos2*: *q* < 0.05; *Il1b*: *p* < 0.0001; *Ptgs2*: *p* < 0.05 vs. CTL-Saline). In contrast, the CFS-HH-205M group showed significantly reduced expression of these inflammatory markers (*Tnf*, *Il6*: *q* < 0.05; *Il1b*: *p* < 0.0001; *Ptgs2*: *p* < 0.05 vs. CFS-Saline), suggesting reduced inflammatory responses.

### HH-205M Treatment Attenuated Intestinal Inflammation

**Fig. 5:**
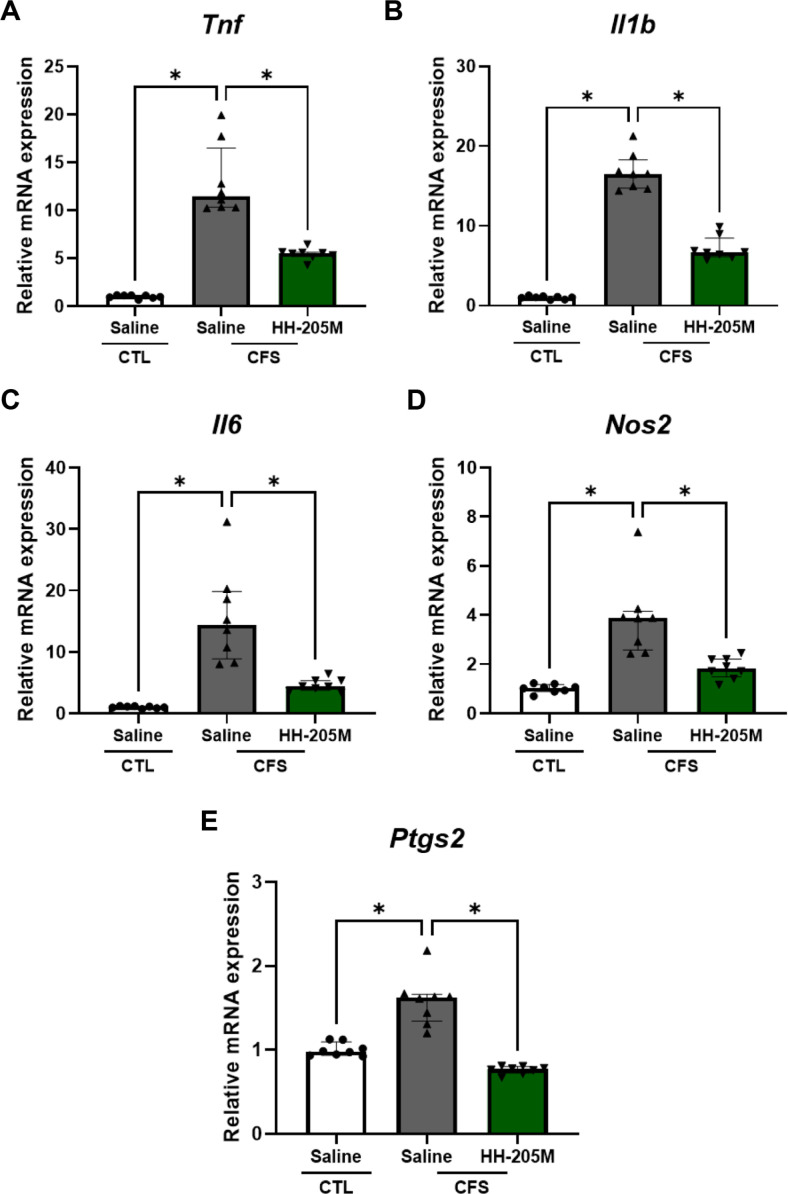
Effect of HH-205M treatment on inflammatory gene expression in colon. (**A–E**) Relative mRNA expression of the pro-inflammatory cytokines, *Tnf*, *Il1b*, and *Il6*, and the pro-inflammatory enzymes, *Nos2* and *Ptgs2*, was determined. All data are presented as the median with IQR (n = 8 per group). Data were analyzed using the Kruskal–Wallis test followed by Dunn’s post-hoc test with Benjamini–Hochberg correction, and asterisks indicate discoveries (*q < 0.05).

Interestingly, CFS-like stress similarly induced inflammatory activation in the colon (Fig. 5A–5E). As observed in muscle, the CFS-Saline group exhibited elevated mRNA expression of inflammatory mediators including *Tnf*, *Il1b*, *Il6*, *Nos2* and *Ptgs2* (*q* < 0.05 vs. CTL-Saline). HH-205M supplementation significantly reduced the expression of these inflammatory markers (*q* < 0.05 vs. CFS-Saline), indicating that HH-205M suppresses CFS-associated inflammatory responses across both skeletal muscle and intestinal tissues.

### HH-205M Remodels the Gut Microbiome and Enriches Barrier-Protective Bacteria

**Fig. 6:**
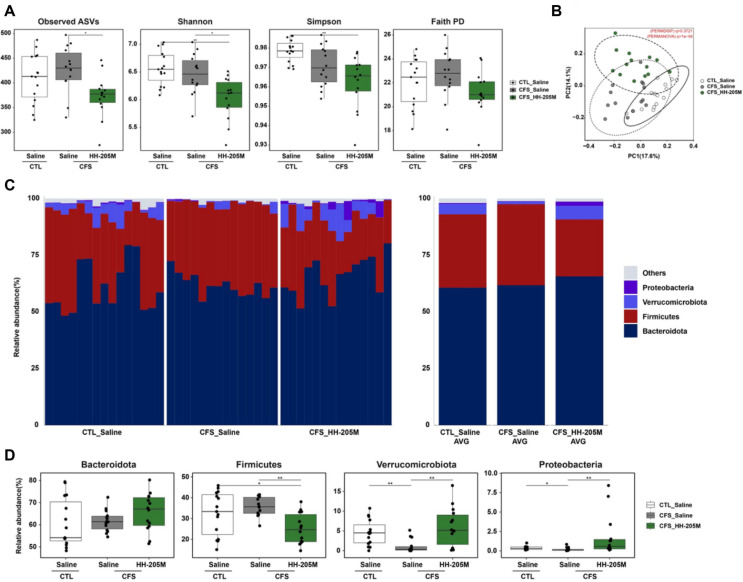
HH-205M treatment modulates gut microbial diversity and reshapes community structure at the phylum level. (**A**) Alpha-diversity indices (Observed ASVs, Shannon, Simpson, and Faith’s PD) indicating bacterial richness and evenness. The decrease in diversity indices in the CFS-HH-205M group reflects the ecological dominance of specific enriched beneficial taxa. (**B**) Principal Coordinate Analysis (PCoA) plot based on Bray–Curtis dissimilarity (*q* = 1 × 10^4^, showing distinct clustering of microbial communities among groups. (**C**) Taxonomic composition at the phylum level. Stacked bar charts visualize the relative abundance of major phyla, illustrating the expansion of Verrucomicrobiota (blue) and Bacteroidota (dark red) in the CFS-HH-205M group. (**D**) Statistical comparison of major phyla. Box plots confirm the increase in Bacteroidota and decrease in Firmicutes, suggesting a shift towards a metabolically favorable Firmicutes/Bacteroidota ratio. In stacked bar charts (**C**), colored segments represent the average relative abundance of taxa. Data in box plots (**A, D**) are presented as median with IQR (n = 14-15 per group). Statistical significance was determined using the Kruskal-Wallis test followed by Dunn's post-hoc test with Benjamini- Hochberg correction (*q < 0.05, **q < 0.01).

To investigate whether the beneficial effects of HH-205M treatment were associated with modulation of the gut microbiota, we analyzed the fecal microbiome using 16S rRNA sequencing (Fig. 6). Alpha-diversity indices (Observed ASVs, Shannon, Simpson, and Faith’s phylogenetic diversity) were significantly lower in the HH-205M-treated CFS group than those in the saline-treated CFS group. Beta-diversity analysis based on Bray–Curtis distances showed distinct separation among the three groups in principal coordinate analysis plots and group differences were supported by permutational multivariate analysis of variance (q = 1 × 10^-4^) without evidence of dispersion heterogeneity (permutational analysis of multivariate dispersions q = 0.3721).

At the phylum level, HH-205M treatment increased the relative abundance of Bacteroidota and Verrucomicrobiota, and decreased that of Firmicutes compared with those in the CFS-Saline group, leading to a lower Firmicutes/Bacteroidota ratio. A concomitant increase in the abundance of Proteobacteria was also observed.

**Fig. 7:**
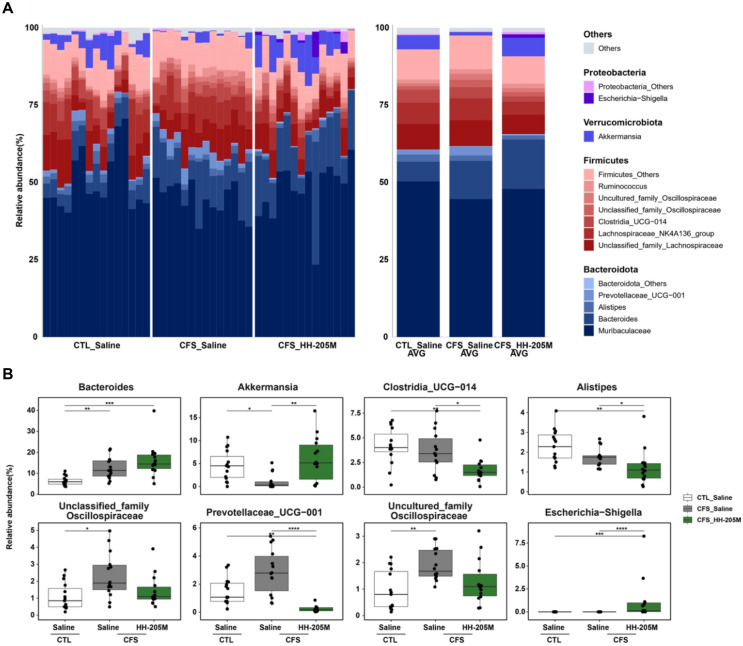
Enrichment of barrier-protective genera and suppression of stress-associated taxa by HH-205M treatment. (**A**) Taxonomic composition at the genus level. Stacked bar charts highlight the distinct microbial profiles, particularly the dramatic bloom of *Akkermansia* (blue segment) and the increase of *Bacteroides* (dark red segment) in the CFS-HH-205M group compared to the CFS-Saline group. (**B**) Statistical comparison of key genera involved in gut barrier function and stress response. HH-205M treatment significantly enriched beneficial taxa (*Akkermansia*, *Bacteroides*) while restoring the levels of stress-associated taxa (*Oscillospiraceae*, *Alistipes*) to those of the control group. In stacked bar charts (**A**), colored segments represent the average relative abundance of taxa. Data in box plots (**B**) are presented as median with IQR (n = 14-15 per group). Statistical significance was determined using the Kruskal-Wallis test followed by Dunn's post-hoc test with Benjamini-Hochberg correction (*q < 0.05, **q < 0.01, ***q < 0.001, ****q < 0.0001).

A detailed taxonomic analysis at the genus level highlighted specific bacterial shifts (Fig. 7). Most notably, the depletion of *Akkermansia*, a mucin-degrading bacterium critical for gut barrier integrity, observed in the CFS-Saline group, was substantially reversed and enriched in the CFS-HH-205M group. Additionally, the abundance of *Bacteroides*, a genus contributing to carbohydrate utilization and the production of short chain fatty acids, such as acetate and propionate, was significantly elevated.

Conversely, the abundance of members of the Oscillospiraceae family, which were elevated in the CFS-Saline group compared with those in the CTL-Saline group, were restored to control levels after HH-205M treatment. Furthermore, *Alistipes* abundance was significantly downregulated in the CFS-HH-205M group compared with that in the CFS-Saline group.

## Discussion

Oxidative stress and systemic inflammation, which are central features of ME/CFS pathophysiology, have frequently been described in patients and are associated with alterations in skeletal muscle energy metabolism [38-40]. Inflammatory stimuli, including endotoxin-related signals such as LPS and stressors such as FSS, are known to induce oxidative stress and inflammatory responses in animal models [41, 42], thereby reproducing ME/CFS-like pathology. These pathological processes promote mitochondrial dysfunction by interfering with critical oxidative pathways, including pyruvate utilization and oxidative phosphorylation, leading to reduced oxidative capacity and metabolic reprogramming toward greater glycolytic reliance [43, 44]. One potential molecular mechanism underlying this process involves upregulation of pyruvate dehydrogenase kinase 4 (PDK4) under inflammatory and oxidative stress conditions, which enhances phosphorylation of pyruvate dehydrogenase (PDH) and suppresses PDH activity [45, 46]. Reduced PDH activity limits pyruvate oxidation and favors a shift toward glycolytic metabolism, thereby increasing lactate production. Although this pathway was not directly assessed in the present study, it may provide a mechanistic framework for understanding inflammation-associated metabolic shifts in the FSS–LPS model.

Such metabolic reprogramming toward glycolytic reliance has been associated with elevated circulating CK, LDH, and lactate, reflecting muscle stress and altered energy metabolism [47, 48]. Lactate accumulation has also been suggested to influence lactate transport and sensing pathways, potentially contributing to fatigue-related phenotypes [49].

In the present study, the FSS–LPS composite model reproduced selected fatigue-like, metabolic, and inflammatory features observed in this study. Serum CK, LDH, and lactate levels were markedly elevated, and skeletal muscle exhibited increased MDA levels, reduced expression of antioxidant enzymes, and upregulation of pro-inflammatory cytokines, consistent with enhanced oxidative and inflammatory stress. Notably, inflammatory activation was observed not only in skeletal muscle but also in colon tissue, suggesting that the FSS–LPS challenge was associated with both local and systemic inflammatory responses. Consistent with evidence that inflammatory conditions could suppress lactate signaling-related genes [50, 51], *Hcar1* and *Slc16a1* expression was downregulated in muscle, which may be linked to altered lactate handling.

These molecular alterations were accompanied by behavioral phenotypes. The CFS–Saline group exhibited shortened latency to exhaustion in the w.l.FST and increased early exhaustion rates in the TMT, indicating reduced physical endurance. These findings are consistent with features such as post-exertional malaise and reduced exercise tolerance, which have been reported in ME/CFS [4].

In contrast, HH-205M administration attenuated the metabolic and inflammatory disturbances induced by FSS–LPS exposure. In the CFS–HH-205M group, circulating CK, LDH, and lactate levels were significantly reduced compared with the CFS–Saline group, accompanied by moderation of oxidative stress markers and pro-inflammatory cytokine expression in skeletal muscle. The anti-inflammatory profile observed in this study aligns with previous reports describing the immunomodulatory properties of HH-205M. Prior studies have demonstrated that HH-205M suppresses LPS-induced NF-κB activation and pro-inflammatory cytokine release in intestinal epithelial cells and reduces circulating inflammatory mediators in murine models of neuroinflammation [52, 53]. Expression of *Hcar1* and *Slc16a1* was also restored following HH-205M treatment. Given the role of these genes in lactate handling, their recovery may indicate improved regulation of lactate-associated metabolic processes under inflammatory stress.

Consistent with these molecular changes, alterations in endurance performance and nociceptive sensitivity were also observed. The CFS–HH-205M group showed prolonged latency to exhaustion and reduced pain hypersensitivity compared with the CFS–Saline group. While direct causal relationships cannot be established, the concurrent improvement in metabolic, inflammatory, and behavioral parameters may suggest a potential association between inflammation-related metabolic modulation and fatigue-like phenotypes in this model.

HH-205M treatment also induced alterations in gut microbiota composition. Although alpha-diversity indices were reduced following HH-205M administration, decreased richness does not necessarily indicate dysbiosis, as probiotic-mediated modulation often involves selective enrichment of specific functionally relevant taxa rather than broad expansion of microbial diversity.

Among the observed changes, enrichment of the genus *Akkermansia* was evident. *Akkermansia muciniphila* is a mucus-associated bacterium that has been linked to intestinal health and immune modulation [54-56]. The enrichment of this genus may be associated with improved mucosal barrier regulation and attenuation of intestinal inflammatory activity, consistent with the reduction in inflammatory markers observed following HH-205M treatment. HH-205M administration also increased the relative abundance of *Bacteroides*, a genus involved in dietary polysaccharide utilization, and the production of short-chain fatty acids (SCFAs), including acetate and propionate [57]. SCFAs are recognized modulators of host immune and metabolic function [58, 59], and recent studies have linked *Bacteroides*-derived propionate to goblet cell differentiation and mucosal maintenance [60]. Although SCFA concentrations were not directly determined, enrichment of SCFA-associated taxa may suggest potential shifts in microbial metabolic capacity, which warrants further investigation.[Fig F8]

Conversely, HH-205M treatment reduced the relative abundance of taxa elevated in the CFS group, including *Alistipes* and members of the Oscillospiraceae family. Increased *Alistipes* abundance has been reported in patients with ME/CFS [61] and in stress-related or depressive conditions [62], and has been associated with alterations in tryptophan metabolism and inflammatory signaling [63]. The reduction of these taxa following HH-205M administration may reflect modulation of stress-associated microbial patterns.

Spearman’s correlation analysis further explored potential associations between gut microbial taxa and metabolic, inflammatory, and behavioral parameters. Notably, *Akkermansia* and the phylum Verrucomicrobiota showed negative correlations with muscle and colon pro-inflammatory markers (*e.g.*, *Il1b*, *Il6*, *Tnf*) as well as lipid peroxidation marker (MDA), while exhibiting positive associations with endurance-related behavioral parameters, including latency to exhaustion. These patterns suggest that enrichment of these taxa may be linked to modulation of inflammatory responses under FSS–LPS-induced stress conditions. Consistent with previous studies reporting that enrichment of *Akkermansia* is associated with reduced systemic inflammation [64, 65], the increase in *Akkermansia* observed in this study may be related to improved systemic inflammatory status. Given the established link between systemic inflammation and skeletal muscle physiology [66, 67], such changes may also be reflected in alterations in muscle inflammatory and oxidative stress responses. In contrast, taxa such as Firmicutes and members of the Oscillospiraceae family tended to display opposite trends, showing positive correlations with inflammatory and lipid peroxidation indicators and negative associations with endurance-related behavioral outcomes and oxidative defense related markers. Although these correlations do not imply causality, they provide additional support for a potential link between microbiota composition and host metabolic and inflammatory status in the FSS–LPS model.

Taken together, the findings of this study suggest that the FSS-LPS composite model captures selected metabolic, inflammatory, and behavioral features relevant to this preclinical fatigue-like context. HH-205M administration moderated these alterations, with concurrent improvements in fatigue-related metabolic markers, inflammatory responses, and behavioral performance. However, it should be noted that the FSS–LPS composite model does not fully capture the complexity of human ME/CFS, and thus direct extrapolation of these findings to clinical conditions should be approached with caution. Within this context, while further mechanistic studies are required to clarify causal relationships, the present results support a potential link between inflammation-associated metabolic stress, gut microbial modulation, and fatigue-like phenotypes in the FSS–LPS model.

## Supplemental Materials

Supplementary data for this paper are available on-line only at http://jmb.or.kr.



## Figures and Tables

**Fig. 8 F8:**
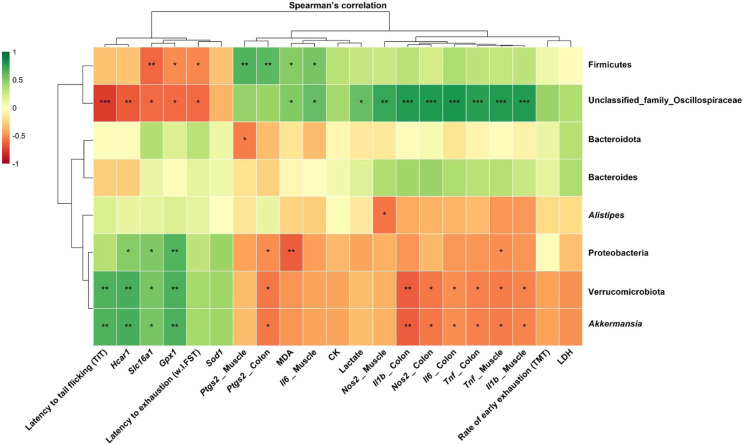
Spearman correlation heatmap between representative gut microbial taxa and fatigue-related molecular and behavioral parameters. Correlation coefficients were calculated using Spearman’s rank correlation analysis. P-values were adjusted for multiple comparisons using the Benjamini–Hochberg correction to control the false discovery rate (FDR). Asterisks indicate statistically significant correlations based on the adjusted values (* *q* < 0.05, **q < 0.01, *** *q* < 0.001). Colors represent correlation coefficients ranging from −1 (red) to +1 (green).
