# Corrigendum to: Preventive Effects of Probiotic Formula on Metabolic Stress Associated Physical Fatigue in Forced Swimming and LPS-Induced Mouse Models

**DOI:** 10.4014/jmb.2026.3606.C06

**Published:** 2026-10-19

**Authors:** Jae Gwang Song, Hyun Jin Bae, Dong Hwan Lee, Joeun Seo, Bomi Lee, Kum-Joo Shin, Eui-Chun Chung, Jeongwook Lee, Hyung Wook Kim, Nam Su Oh

**Affiliations:** 1 Department of Integrative Bioscience and Biotechnology, Sejong University, Seoul 05006, Republic of Korea; 2 Department of Food and Biotechnology, Korea University, Sejong 30019, Republic of Korea; 3 R&D Center, Hecto Healthcare Co. Ltd., Seoul 06142, Republic of Korea

In the article titled “Preventive Effects of Probiotic Formula on Metabolic Stress Associated Physical Fatigue in Forced Swimming and LPS-Induced Mouse Models,” the following grant information was inadvertently omitted from the Funding section of the article. The correct funding statement should read as follows:

**This work was supported by the Ministry of Science and ICT (MSIT), Korea, under the Information Technology Research Center (ITRC) support program (grant number IITP-2025-RS-2023–00258971); and supervised by the Institute for Information & Communications Technology Planning & Evaluation (IITP) and Hecto Healthcare Co., Ltd. This work was also supported by a Korea University Grant (grant number K2213531).**

