# Supplementary material for: Preventive Effects of Probiotic Formula on Metabolic Stress Associated Physical Fatigue in Forced Swimming and LPS-Induced Mouse Models
Source: J Microbiol Biotechnol. 2026 Apr 10;36:e2603034. doi: 10.4014/jmb.2603.03034 (PMC13070968; doi:10.4014/jmb.2603.03034)
Supplement: Supplementary file 1 [file jmb-36-e2603034-supple.pdf]

J. Microbiol. Biotechnol. 2026. 36: e2603034
https://doi.org/10.4014/jmb.2603.03034

Preventive Effects of Probiotic Formula on Metabolic Stress Associated Physical Fatigue in Forced Swimming and LPS-Induced Mouse Models

Jae Gwang Song†[1], Hyun Jin Bae†[2], Dong Hwan Lee†[2], Joeun Seo[1], Bomi Lee[1], Kum-Joo Shin[3], Eui-Chun Chung[3], Jeongwook Lee[3], Hyung Wook Kim*[1], Nam Su Oh*[2]*

[1]Department of Integrative Bioscience and Biotechnology, Sejong University, Seoul 05006, Republic of Korea

[2]Department of Food and Biotechnology, Korea University, Sejong 30019, Republic of Korea

[3]R&D Center, Hecto Healthcare Co. Ltd., Seoul 06142, Republic of Korea

†Jae Gwang Song, Hyun Jin Bae, and Dong Hwan Lee contributed equally to this study.

*** Corresponding author: Hyung Wook Kim and Nam Su Oh**

E-mail: kimhyung@sejong.ac.kr and klanvin@korea.ac.kr

Supplementary Table S1. Primer sequences used for amplification

Gene	Forward Primer (5' to 3')	Reverse Primer (5' to 3')
Hcar1	ATCCTGGTCTTCGTGCTTGG	CTGTCCGAAGGGGTAAGCAG
Slc16a1	GACCATTGTGGAATGCTGCCCT	CGATGATGAGGATCACGCCACA
Tnf	CTGTAGCCCACGTCGTAGC	TTGAGATCCATGCCGTTG
Il1b	TGCCACCTTTTGACAGTGATG	TTGGAAGCAGCCCTTCATCTT
Il6	TAGTCCTTCCTACCCCAATTTCC	TTGGTCCTTAGCCACTCCTTC
Nox2	CCCTTCCGAAGTTTCTGGCAGCAGC	GGCTGTCAGAGAGCCTCGTGGCTTTGG
Ptgs2	TGTATCCCCCCACAGTCAAAGACAC	GTGCTCCCGAAGCCAGATGG
Sod1	GGTCTCCAACATGCCTCTCT	AACCATCCACTTCGAGCAGA
Gpx1	TACACCGAGATGAACGATCTG	ATTCTTGCCATTCTCCTGGT
Gapdh	CATCACTGCCACCCAGAAGACTG	ATGCCAGTGAGCTTCCCGTTCAG
